# Assessment of the liver with two‐dimensional shear wave elastography following COVID‐19 infection: A pilot study

**DOI:** 10.1002/ajum.12390

**Published:** 2024-05-06

**Authors:** Joyce Yea See Lau, Sandra O'Hara, Paul Lombardo, Melinda Goodyear

**Affiliations:** ^1^ SKG Radiology Level 3, 1 Hood Street Subiaco 6008 Western Australia Australia; ^2^ Department of Medical Imaging and Radiation Sciences Monash University Wellington Rd Clayton 3800 Victoria Australia; ^3^ School of Rural Health Monash University Wellington Rd Clayton 3800 Victoria Australia

**Keywords:** COVID‐19, elastography, liver, liver injury, shear wave elastography, ultrasound

## Abstract

**Introduction/Purpose:**

The coronavirus disease (COVID‐19) is a widely spread viral infectious disease, which can impact multiple organs, including the liver. Elevated liver enzymes have been reported in COVID‐19 patients; however, potential changes in liver stiffness following the viral infection remain uncertain. The main aim of this pilot study was to determine if there is a significant difference in liver stiffness between individuals who have never been infected with COVID‐19 and those who had been infected with COVID‐19 <6 months, experiencing only mild symptoms. The secondary aim was to compare the liver stiffness between participants infected with COVID‐19 depending on the elapsed time since infection.

**Methods:**

Two‐dimensional shear wave elastography (2D‐SWE) was performed prospectively on 68 participants. Thirty‐four participants had been infected with COVID‐19 (all for <6 months) (COVID‐19 group), and another 34 had never been infected with COVID‐19 (control group). The mean 2D‐SWE measurements of both the COVID‐19 group and the control group were compared using an independent *t*‐test. The mean 2D‐SWE measurements of the COVID‐19 subgroups A (<2 months), B (2 to <4 months) and C (4 to <6 months) were compared using a one‐way ANOVA test (P < 0.05).

**Results:**

The (mean ± standard deviation) liver stiffness (kPa) of the COVID‐19 group (5.26 ± 1.63 kPa) was significantly higher than the control group (4.30 ± 0.96 kPa) (P = 0.005). There was no significant difference in liver stiffness among subgroups A (5.20 ± 1.79 kPa), B (4.70 ± 1.53 kPa) and C (5.96 ± 1.48 kPa) (P = 0.143) respectively.

**Discussion:**

The mean liver stiffness of 4.30  ±  0.96k Pa in the control group showed a high probability of being normal as per guidelines. Conversely, the mean liver stiffness of 5.26  ±  1.63 kPa in the COVID‐19 group exhibited a statistically significant increase compared to the control group. However, compensated advanced chronic liver disease was ruled out without other known clinical signs, as per guidelines.

**Conclusion:**

A statistically significant increase in liver stiffness value was observed in the post‐COVID‐19 infection group compared to the group who had never been infected. This highlights the potential for short‐term impact on liver stiffness associated with COVID‐19 infection. However, it is unclear if these changes in liver stiffness are associated with liver injury. Further study is warranted to investigate the effects of COVID‐19 infection and its long‐term impact on the liver.

## Introduction

The coronavirus disease (COVID‐19) is a widely spread viral infectious disease, caused by severe respiratory syndrome coronavirus 2 (SARS‐COV‐2) virus.^1^ Although the virus SARS‐COV‐2 is known to primarily cause respiratory diseases such as pneumonia or acute respiratory distress syndrome (ARDS), evidence suggests that SARS‐COV‐2 can also affect multiple organs, including the liver.^2^ A recent meta‐analysis concluded that COVID‐19‐induced liver injury is generally minimal, but can be more significant in severe cases, potentially causing hepatocellular injury, liver metabolic and synthetic function abnormalities.^3^ These may be due to the severe inflammatory response, binding of SARS‐COV‐2 to the target liver cells, or drug treatments, and can be observed in those with or without pre‐existing liver conditions.^4^

The angiotensin‐converting enzyme 2 (ACE2) receptor plays an important role in COVID‐19 viral transmission.^5^ These receptors can be detected in the liver and are more highly expressed in cholangiocytes than hepatocytes.^6^ Cholangiocytes can be found in bile duct epithelial cells, whereas hepatocytes can be found in Kupffer cells in the liver.^7^ Thus, apart from directly inducing bile duct abnormalities, SARS‐COV‐2 can also cause cholestasis by infecting liver cells through the biliary epithelial cells.^8^, ^9^ Significant elevations of liver enzymes such as alanine aminotransferase (ALT), aspartate aminotransferase (AST), gamma‐glutamyl transferase (GGT) and bilirubin have been reported in COVID‐19 patients.^10^, ^11^, ^12^, ^13^, ^14^ The level of elevation of AST, ALT and bilirubin is related to the severity of COVID‐19 symptoms, while GGT was found to be three times higher than the normal limit in COVID‐19 patients.^13^, ^14^, ^15^

Liver injury can also be caused by the prescribed medications for COVID‐19 treatment. Patients who received ribavirin, corticosteroids, antibiotics, antifungal drugs, immunotherapy or parenteral and enteral nutrition during their infectious period were found to have drug‐induced liver injury.^16^ The use of other medications for COVID‐19 treatments such as remdesivir (Veklury®), lopinavir/ritonavir (Kaletra®) and favipiravir (Avigan®) has also been reported to cause abnormal liver enzymes.^13^, ^17^, ^18^, ^19^

Evidence also suggests patients who have pre‐existing liver disease are at higher risk of liver damage following a COVID‐19 infection.^4^ There is an increased risk of severe COVID‐19 infection in cirrhosis patients and subsequent deterioration into advanced cirrhosis and acute liver failure.^20^ COVID‐19 patients with chronic liver diseases such as cirrhosis, hepatitis B virus (HBV), hepatitis C virus (HCV), alcoholic liver disease (ALD), non‐alcoholic fatty liver disease (NAFLD) or hepatocellular carcinoma (HCC) have higher mortality compared to those without chronic liver disease.^21^

Liver fibrosis results from damage to the liver by increasing the deposition of the extracellular matrix to promote the healing process.^22^ Traditionally, liver biopsy has been the gold standard for investigating the level of fibrosis caused by viral infections in the liver. However, it comes with risks and complications to the patient such as sampling error, bleeding and death.^22^ Alternatively, 2D‐SWE provides a non‐invasive method to evaluate liver stiffness and indirectly evaluate the level of liver fibrosis.^23^ 2D‐SWE can be used to detect the stiffness of liver tissue by delivering the modified acoustic radiation force impulse (ARFI) to displace the tissue in the region of interest.^24^ Shear waves can be produced and propagated faster in stiffer liver tissue and slower in softer liver tissue.^24^ These movements of shear waves can be tracked in a similar method to that used by Doppler ultrasound technology.^24^ This low‐risk, non‐invasive method provides high sensitivity and specificity for evaluating liver fibrosis and cirrhosis.^23^, ^25^, ^26^, ^27^

Potential liver changes following the SARS‐COV‐2 viral infection remain unclear as there are conflicting findings from a limited number of studies assessing liver stiffness after a COVID‐19 infection.^28^, ^29^ Most of the participants in these studies were hospitalised and presented with moderate‐to‐severe symptoms such as pneumonia and acute respiratory distress syndrome (ARDS).^28^, ^29^ Pre‐existing liver diseases such as fatty liver and viral hepatitis were also reported in the participants of these studies.^28^, ^29^ The presence of these coexisting conditions may increase liver stiffness in these participants.^30^ Potential liver changes in people who had been infected with COVID‐19 but experienced mild symptoms have not been explored in the published literature.

This research aims to investigate the changes in liver stiffness following COVID‐19 infection with mild symptoms using 2D‐SWE technology. The main aim is to compare the liver stiffness between individuals who have never been infected with COVID‐19 and those who have been infected with mild symptoms for up to 6 months. The secondary aim is to compare the liver stiffness among individuals who have been infected with COVID‐19 within the last 6‐months during the study recruitment period.

## Materials and methods

### Participants

A prospective case–control study was conducted at branches of SKG Radiology in Perth, Western Australia, from December 2022 to April 2023. Participants were recruited from SKG Radiology staff and patients who attended a branch for an upper abdominal ultrasound examination. This study was limited to participants between 18 and 60 years of age. Staff participants were recruited by email invitation from the administration staff who were not involved in the research. Patient participants were invited to participate when attending a routine abdominal ultrasound examination at branches of SKG Radiology. All participants were required to read an explanatory statement and provide informed written consent prior to being enrolled in the study.

All participants fasted for a minimum of 6 hours and abstained from alcohol for 12 hours prior to the examination. All participants provided information if they had been previously diagnosed with chronic liver diseases such as viral hepatitis, NAFLD, ALD and cirrhosis. This study excluded participants with pre‐existing liver conditions, as well as those who were presented with incidental findings in the region of interest (ROI) during the 2D‐SWE examinations.

Participants in the COVID‐19 group provided details of the time and severity of infection. Mild symptoms were general respiratory symptoms such as fever, cough and a runny nose. Symptoms were deemed severe if the participant was hospitalised or attended an emergency department during the infectious period. For data analysis, the participants were divided into three subgroups: <2 months post‐infection (subgroup A), 2 to <4 months post‐infection (subgroup B) and 4 to <6 months post‐infection (subgroup C).

### Ultrasound imaging

**Figure 1:**
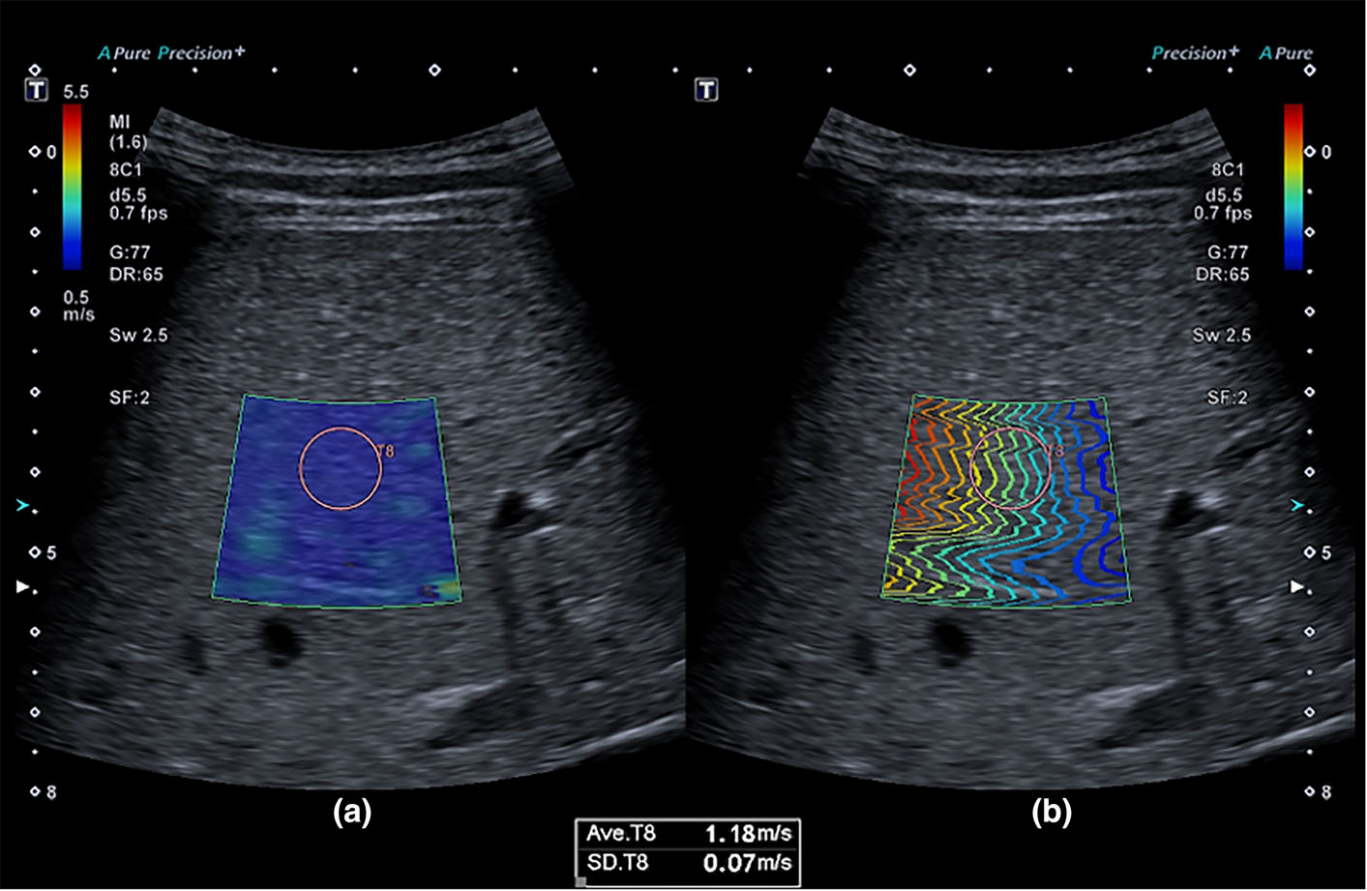
Example of two‐dimensional shear wave elastography (2D‐SWE) with the placement of the circular region of interest (ROI). The circular ROI is placed in the elastogram (a) that has the most uniform colour which is concordant with the circular ROI within the wavefront map (b) showing the most parallel and equidistant propagation lines. This ROI will output the shear wave values with the lowest standard deviation.

Two‐dimensional shear wave elastography ultrasound imaging was performed using Canon a550 ultrasound machines (Otawara, Tochigi, Japan). The elastogram was set to a box size of 2.5 × 3 cm with a 10 mm circular region of interest (ROI) used to obtain quantitative shear wave measurements reported in kilopascal (kPa)s and meters per second (shear wave speed) (Figure 1).

Two‐dimensional shear wave elastography measurements were performed as per international guidelines.^23^, ^31^ Participants were placed in the supine position with slight right arm extension.^23^ Measurements were acquired with an intercostal ultrasound approach and a neutral breath hold. ROI placement was at a minimum depth of 1.5–2 cm below the liver capsule in segment 5, 7 or 8 (Figure 1).^23^ The ROI was placed within the elastogram region with the most uniform colour and the wavefront map region with the most parallel, equidistant propagation lines and lowest standard deviation (SD) (Figure 1).^31^ Measurements reported in metres per second with an SD value of >20% of the mean^31^ or measurements reported in kPa(s) with an SD of >30% of the mean^23^ were considered inaccurate and excluded from the data set.

As per established guidelines, a minimum of five accurate liver stiffness measurements were required for each participant to formulate the reportable median shear wave values in speed and kPa(s).^23^ The reliability of the data set was assessed using the interquartile range and median ratio (IQR/M) ×100. This is required to be ≤30% for measurements reported in kPa and ≤15% for measurements reported in metres per second (m/s) for the data set to be considered accurate.^23^

Data were acquired by Australian Sonographer Accreditation Registry (ASAR)‐accredited qualified sonographers with >5 years of experience in the field of ultrasound and were experienced in the use of 2D‐SWE. Intra‐operator testing was not performed in this study as 2D‐SWE has already been shown to perform with high reproducibility.^32^, ^33^, ^34^ Due to the limitation of the research design, the sonographers were not blinded to the COVID‐19 status of participants during data collection. Nonetheless, strict adherence to international guidelines was maintained to mitigate bias.^23^, ^31^

### Statistical analysis

The median shear wave values were collected from each participant as per established guidelines.^23^ The collected data were analysed using IBM SPSS version 29.0. Normality testing was performed by the Shapiro–Wilk test due to the small sample size, and the assumption of normality was met. Subsequently, the mean value for each group was calculated for group comparison.

An independent *t*‐test was performed to compare the overall mean liver stiffness obtained from the control and COVID‐19 groups. The null hypothesis was that the mean liver stiffness in the COVID‐19 group was equal to the mean liver stiffness in the control group, which was operationalised as the paired differences in kPa with a posited mean of 0, tested against a two‐sided alternative, at the 5% level of statistical significance (P < 0.05).

Additionally, a one‐way ANOVA test was used to analyse the differences in mean liver stiffness of participants in subgroups A, B and C. The null hypothesis used was that the mean liver stiffness in subgroup A was equal to the mean liver stiffness in subgroup B, and both were equal to the mean liver stiffness in subgroup C, tested at the 5% level of statistical significance (P < 0.05). This hypothesis stated that there were no differences in mean liver stiffness regardless of the time elapsed since infection.

### Ethics approval

Ethics approval was obtained from the SKG Radiology Institutional Review Board and the Monash University Human Research Ethics Committee (Project ID: 36372).

## Results

A total of 70 individuals initially met the eligibility criteria for participation. Two individuals were excluded due to the finding of a liver mass in the ROI during the 2D‐SWE examination. Of the remaining 68 participants, 39 were staff participants and 29 were patient participants. The gender distribution comprised 14 males and 54 females, with an average age of 41.1 years. The demographic details are presented in Table 1.

**Table 1:** Demographic characteristics of study participants.

	Mean age (Years)	Male (n)	Female (n)	Total participants (n)
Control group	45.0	7 (20%)	27 (80%)	34
COVID‐19 group	37.4	7 (20%)	27 (80%)	34
Subgroup A	35.0	2 (25%)	6 (75%)	8
Subgroup B	38.0	3 (21%)	11 (79%)	14
Subgroup C	38.1	2 (17%)	10 (83%)	12

**Figure 2:**
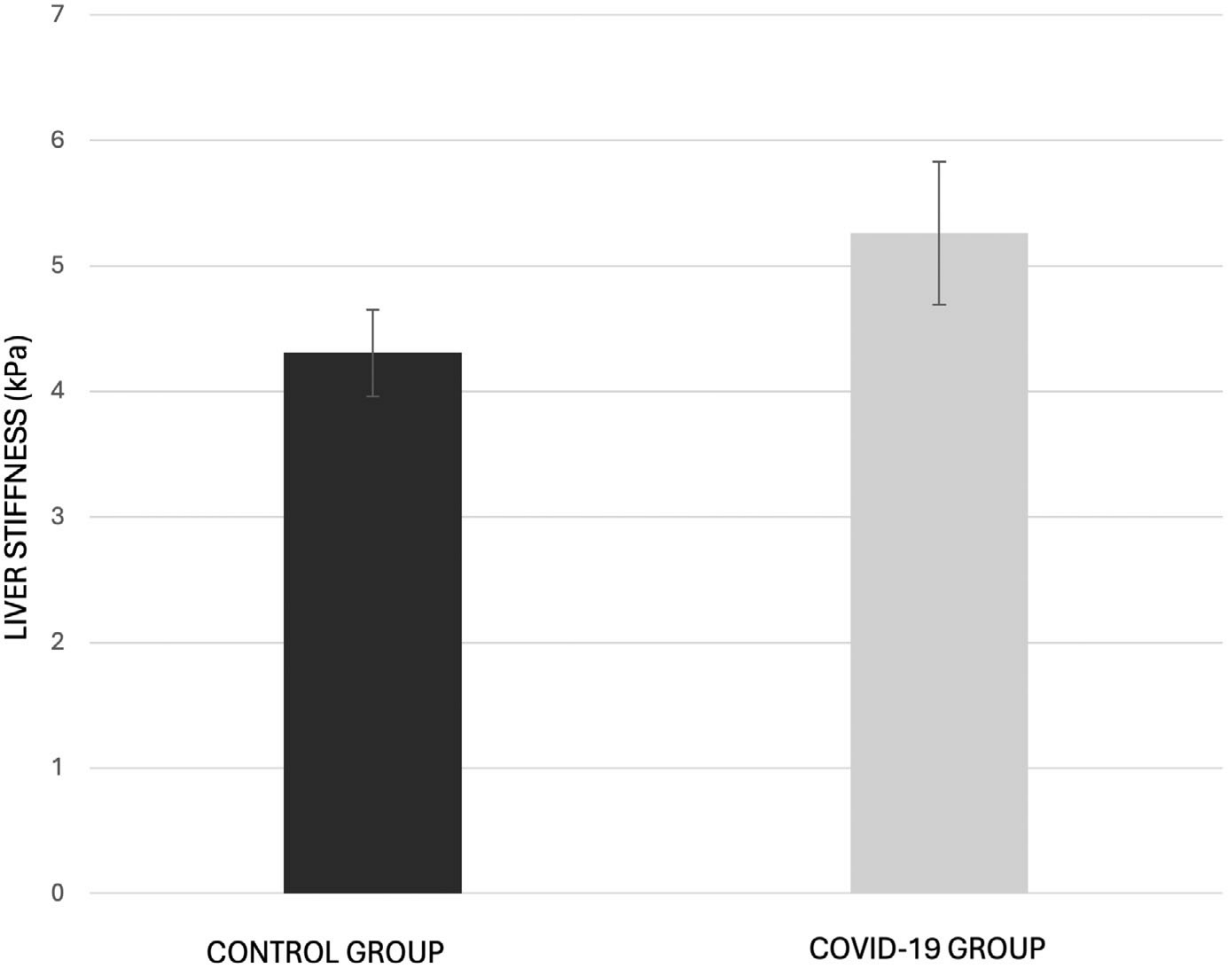
Comparison of mean liver stiffness for the control group and the COVID‐19 group. Error bars represent the standard error of the mean. The COVID‐19 group (5.26 ± 1.63 kPa) shows a significantly higher increase in mean liver stiffness compared to the control group (4.30 ± 0.96 kPa) [*t*(53.69) = −2.941, P = 0.005].

The mean (±SD) of liver stiffness was found to be significantly lower in the control group (4.30 ± 0.96 kPa) compared to the mean liver stiffness in the COVID‐19 group (5.26 ± 1.63 kPa) (Figure 2). The results revealed a *t*‐value of −2.941 (df = 54, P = 0.005) indicating a statistically significant difference in liver stiffness between the two groups.

**Figure 3:**
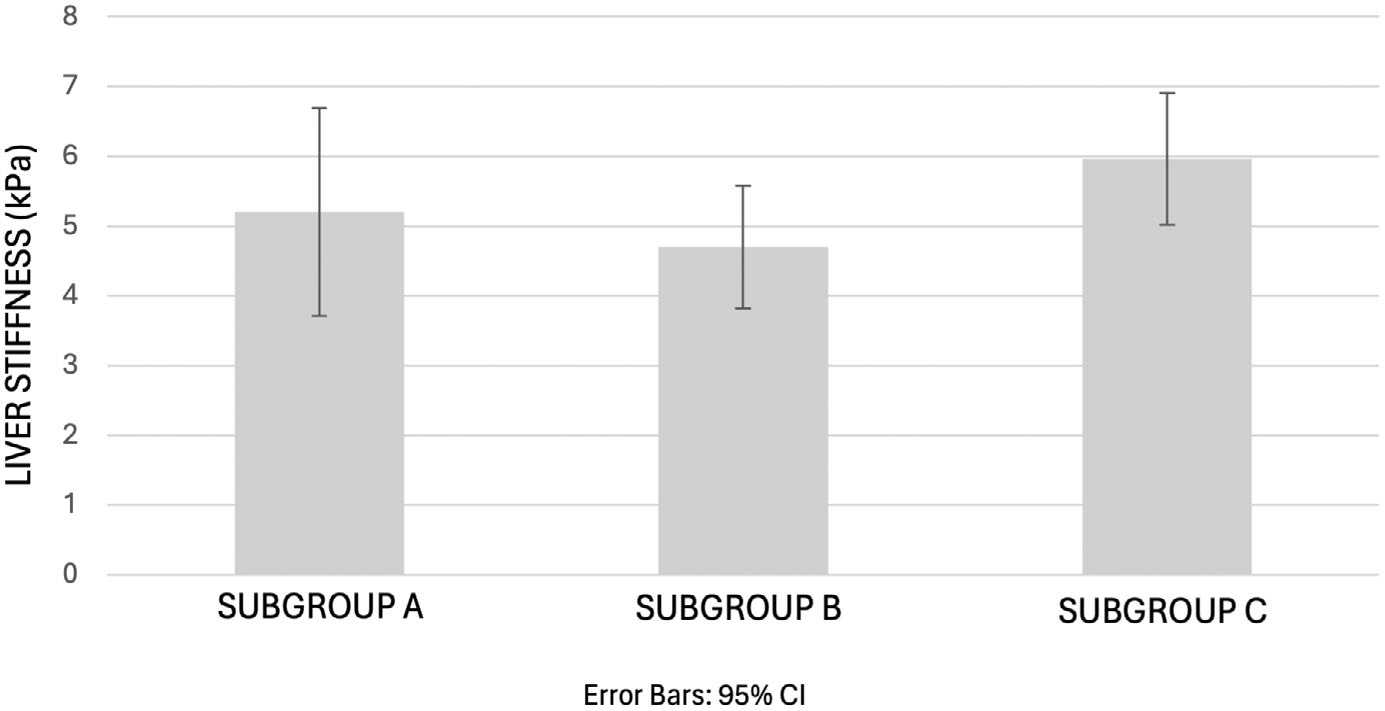
Comparison of mean liver stiffness among three COVID‐19 subgroups A, B and C. Participants from subgroup A were <2 months post‐infection, participants from subgroup B were within 2 to <4 months post‐infection and participants from subgroup C were within 4 to <6 months post‐infection. Error bars represent the standard error of the mean. There was no statistically significant difference in mean liver stiffness among all three subgroups (P = 0.143, ANOVA).

The one‐way ANOVA test revealed that there was not a statistically significant difference in mean liver stiffness between all three subgroups A, B and C (*F* (2, 31) = [2.072], P = 0.143). The mean ± standard deviation of liver stiffness in subgroup A (n = 8), subgroup B (n = 14) and subgroup C (n = 12) was 5.20 ± 1.79, 4.70 ± 1.53 and 5.96 ± 1.48 kPa, respectively (Figure 3).

## Discussion

The results of this study showed a statistically significant difference in liver stiffness in participants who had a mild COVID‐19 infection in the 6 months prior to the 2D‐SWE examination, compared to those who had never been infected. According to the established guideline,^23^ liver stiffness of 5 kPa or less has a high probability of being normal; liver stiffness <9 kPa, in the absence of other known clinical signs, rules out compensated advanced chronic liver disease (cACLD); liver stiffness between 9 kPa and 13 kPa are suggestive of cACLD but may need a further test for confirmation; and liver stiffness > 13 kPa is highly suggestive of cACLD. The mean liver stiffness obtained from this study for the COVID‐19 group was 5.26 (±1.63) kPa, which was <9 kPa. Although this ruled out cACLD in the absence of other known clinical signs in our COVID‐19 group, this was still a statistically significant increase compared to the control group of patients who had never been infected.

To date, there has been limited published work assessing liver stiffness following COVID‐19 infection using 2D‐SWE, with conflicting results. A study by Kolesova *et al*.^29^ compared the liver stiffness of 58 individuals who had been infected with COVID‐19 infection (post‐COVID‐19 group) to 17 individuals without COVID‐19 (control group). Their study revealed no significant differences between the post‐COVID‐19 (4.60 kPa [IQR: 4.00, 5.53]) and the control group (4.55 kPa [IQR: 4.15, 4.88]). Individuals in the control group had no history of chronic liver disease, but 3% of participants in the COVID‐19 group had chronic liver disease.^29^ Participants in the COVID‐19 group were 3–6 months post‐acute infection, and were hospitalised with severe symptoms such as pneumonia, decreased blood oxygen and acute respiratory distress syndrome (ARDS).^29^ Another study from Radzina *et al*.^28^ analysed 90 individuals: 56 individuals who had been infected with COVID‐19, 3–9 months prior to enrolment, and 34 individuals who served as the healthy control group. This study revealed a statistically significant increase in 2D‐SWE values in the COVID‐19 group (5.06 ± 1.69 kPa) compared to the healthy group (4.56 ± 0.90 kPa) (P < 0.001).^28^ In the COVID‐19 group, 60% of participants were hospitalised, and 56% had moderate‐to‐severe symptoms, including pneumonia and ARDS.^28^ There were 17% of participants from the COVID‐19 group and 12% of participants from the control group that had pre‐existing liver conditions.^28^

Our work excluded individuals who were previously diagnosed with chronic liver diseases such as AFLD, NAFLD, HBV, HBC and cirrhosis. Chronic liver diseases result in liver fibrosis, altering the stiffness of the liver and potentially increasing 2D‐SWE measurements.^30^ This exclusion aimed to minimise the impact of pre‐existing liver diseases on the 2D‐SWE measurements obtained for this work. This work was also focused on individuals with mild symptoms. This aimed to reduce the impact of complications due to severe COVID‐19 symptoms, such as drug‐induced liver injury. Hospitalised COVID‐19 patients with severe symptoms often require intensive drug treatments, which potentially cause liver injury.^13^, ^17^, ^18^, ^19^ Our results are similar to those obtained by Radzina *et al*., who reported that the mean liver stiffness of individuals who have been infected with COVID‐19 is significantly higher than the healthy control group. Interestingly, this has occurred even though there is a difference in participant symptomology between these groups.

To further investigate the changes in liver stiffness in post‐COVID‐19 infection, our study also compared the mean liver stiffness within the COVID‐19 group depending on the time since infection. The liver holds the ability for complete self‐repair, allowing damaged or fibrotic liver the potential for reversal and regeneration.^35^ Our work did not show a statistically significant difference in liver stiffness between participants who were infected <2, 2 to <4 and 4 to <6 months prior to the 2D‐SWE examination. This result was limited by the small number of participants in each group. Further studies are warranted with larger sample sizes and an extended timeframe. Longer intervals may be needed to compare post‐COVID‐19 infection liver stiffness. Considering follow‐up 2D‐SWE for each participant at 6, 12, 18 and 24 months post‐infection, with each participant becoming his or her control, may provide valuable insights into changes in liver stiffness over time.

There were some limitations to this work. All participants in this study were self‐reported as healthy and had not been diagnosed with liver diseases. Without other confirmatory blood or medical examinations, there may have been a potential for undetected liver disease in both groups. There is also the potential for undiagnosed cases of COVID‐19 to be present in the control group. Ideally, a liver function blood test and serology antibody tests for SARS‐CoV‐2 can be obtained prior to the 2D‐SWE examination to exclude pre‐existing liver conditions and undiagnosed COVID‐19 infection in any future studies.

## Conclusion

In conclusion, a statistically significant increase in liver stiffness values was observed in individuals up to 6 months post‐COVID‐19 infection with mild symptoms compared to individuals who had never been infected. This highlights the potential for short‐term impact on liver stiffness associated with COVID‐19 infection. However, it is unclear if these changes in liver stiffness are associated with liver injury. Future direction for this work could involve an extended research duration to obtain a larger sample size and a more robust understanding of the effect of COVID‐19 infection and its long‐term impact on the liver.

## Author contributions

**Joyce Yea See Lau:** Conceptualization, Investigation, Writing – original draft, Methodology, Validation, Visualization, Software, Formal analysis, Project administration, Data curation. **Sandra O'Hara:** Conceptualization, Investigation, Methodology, Validation, Writing – review & editing, Formal analysis, Data curation, Supervision, Resources. **Paul Lombardo:** Conceptualization, Methodology, Validation, Writing – review & editing, Supervision, Resources. **Melinda Goodyear:** Supervision, Formal analysis, Methodology, Validation, Writing – review & editing.

## Funding

No funding information is provided.

## Conflict of interest

The authors have no known conflict of interest to disclose.
